# Triple mapping for axillary staging after neoadjuvant therapy: Axillary reverse mapping with indocyanine green and dual agent sentinel lymph node biopsy

**DOI:** 10.1097/MD.0000000000032545

**Published:** 2022-12-30

**Authors:** Ilhan Tasdoven, Hakan Balbaloglu, Rabiye Uslu Erdemir, Burak Bahadir, Cakmak Guldeniz Karadeniz

**Affiliations:** a Zonguldak Bulent Ecevit University, School of Medicine, Department of General Surgery, Zonguldak, Turkey; b Zonguldak Bulent Ecevit University, School of Medicine, Department of Nuclear Medicine, Zonguldak, Turkey; c Zonguldak Bulent Ecevit University, School of Medicine, Department of Clinical Pathology, Zonguldak, Turkey.

**Keywords:** axillary reverse mapping, indocyanine green, isosulfan blue, lymphedema, neoadjuvant systemic therapy, sentinel lymph node biopsy

## Abstract

Axillary staging is 1 of the major issues of current breast cancer management after neoadjuvant systemic therapy (NST). Sentinel lymph node biopsy (SLNB) is an option for clinically node negative patients. Axillary reverse mapping (ARM) was introduced to identify and preserve the lymphatic drainage from the arm. The aim of the presented study is to employ triple mapping (radiocolloid, blue dye and indocyanine green [ICG]) to assess the crossover rate and metastatic involvement of ARM nodes after NST. Clinically node positive patients before NST who were converted to N0 and scheduled for targeted axillary dissection were included. sentinel lymph node (SLN) mapping was performed via dual agent mapping. ICG was used for ARM procedure. Blue, hot and fluorescent nodes and lymphatics were visualized in the axilla using infrared camera system and dual opto-nuclear probe (Euoroprobe3). Fifty-two patients underwent targeted axillary dissection and ARM procedures 12 out of whom had axillary node dissection. 45 of the 52 patients had at least 1 hot or blue SLN identified intraoperatively. Of these, 61.5% cases had hot SLNs, 42.3% had hot and blue, 15.4% had hot/blue/fluorescent, 7.7% had blue/fluorescent, 6 11.5% had hot/fluorescent and 7 13.5% had only clipped nodes. The overall identification rate of ARM-nodes by means of ICG technique was 86.5%. Overall crossover of ARM nodes with SLNs was determined in 36.5%. The ICG intensity was found to be higher in both hot and blue SLNS (8 out of 18 ICG positive cases, 44.4%). In 3 of 52 patients (5.7%) metastatic SLNs were hot or blue but fluorescent which predicts metastatic involvement of the ARM-nodes. More than 1-third of the patients revealed a crossover between arm and breast draining nodes. The higher observed rate of overlap might partially explain why more patients develop clinically significant lymphedema after NST even after sentinel lymph node biopsy alone. The triple mapping provides valuable data regarding the competency of lymphatic drainage and would have the potential to serve selecting patients for lymphovenous by-pass procedures at the index procedure. NST reduces the metastatic involvement of the ARM nodes. However, conservative axillary staging with sparing ARM nodes after NST necessitates further studies with larger sample size and longer follow-up.

## 1. Introduction

The evaluation of the axillary status is 1 of the major issues of current breast cancer management after neoadjuvant systemic therapy (NST). Fortunately, Sentinel lymph node biopsy (SLNB) replaced conventional complete axillary lymph node dissection (ALND) for clinically node negative patients after NST with lower lymphedema rates ranging up to 13%.^[1–3]^ Axillary reverse mapping (ARM) was introduced to identify and preserve the lymphatic drainage from the arm to solve the dilemma between oncologic safety and arm morbidity with the hypothesis of 2 distinct lymphatic pathways draining the breast and upper extremities, which are interconnected anatomically.^[4–8]^ Currently, the conventional dual agent mapping is the standard of care for sentinel lymph node (SLN) detection after NST to decrease false negative rates and includes 99mTc-labeled radiotracer and isosulfan blue dye combination with various limitations.^[9–12]^ Therefore, a third agent for ARM procedure was required and a fluorescent method using indocyanine green (ICG) has been developed.^[13]^ The sensitivity of this imaging technique is very high for the identification of ARM nodes and allows to evaluate the crossover between blue and hot SLNs and fluorescent ARM nodes.^[14]^ The most crucial point is the crossover between arm and breast sentinel nodes ranging up to 28%.^[15,16]^ Therefore, we sought for a simple and safe method to detect SLN with dual agent mapping and reserve ARM nodes identified with ICG, for patients who converted to clinically nod negative after NST. The purpose of this study was to assess the crossover rate and involvement of ARM nodes in patients with clinically node negative disease after NST who underwent dual agent SLNB.

## 2. Methods

### 2.1. Study cohort

Between January 2018 and January 2020, female patients aged over 18 years presenting with clinically node positive, invasive breast cancer, who converted to clinically node negative after NST and scheduled for targeted axillary dissection (TAD) (clipped node + SLNB) were included in the study. Exclusion criteria were adverse events during the TAD procedure, pregnancy, persistence of clinically positive status, a history of breast cancer, previous axillary surgery, and distant metastasis. All patients received NST in the form of chemotherapy; none of the patients received neoadjuvant endocrine therapy according to the multidisciplinary board recommendations and were informed about the potential risks and advantages of the study. A signed written informed consent for participation was achieved. The complete axillary clinical response after NST was defined as disappearance of metastatic features on physical examination, ultrasound, magnetic resonance imaging, or positron emission tomography-computer tomography imaging. Patients with suspicion of persistent axillary disease including cortical thickening > 2 mm, loss of fatty hilum, matted nodes on physical examination, and marked hypoechogenisity in the cortex after NST were excluded and underwent ALND.

### 2.2. TAD and ARM procedure

**Figure 1.**
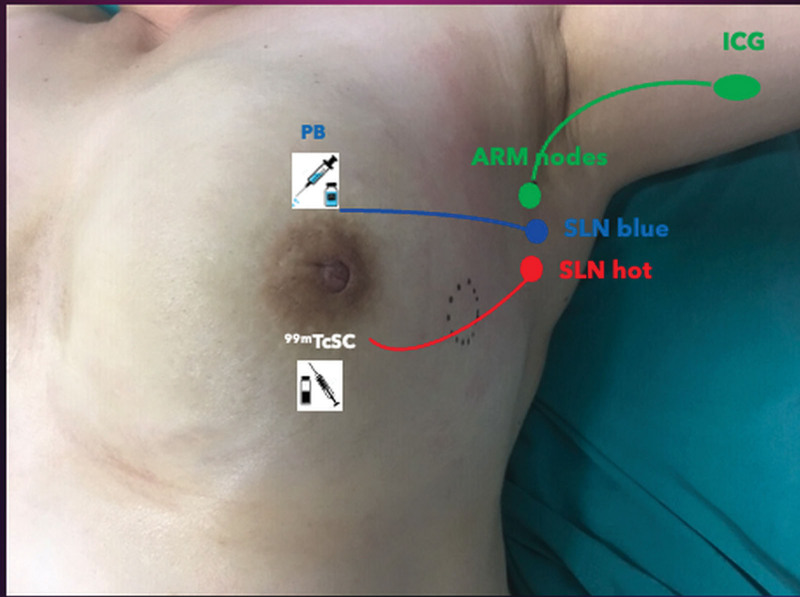
The triple mapping technique.

In each case, cytologically positive nodes were clipped prior to NST. The injection of 99M-technetium-sulfur colloid was performed circumferential to the nipple areola complex, at 4 points and followed by lymphoscintigraphy. In the operation theater, the ARM-procedure was carried out via injection of 1 to 3 mg/mL ICG subcutaneously in the medial aspect of the ipsilateral proximal arm, at the intermuscular groove between the biceps and triceps muscles which have been defined previously.^[4,17]^ Subsequently, the injection site was massaged gently for 5 to 7 minutes. At the same time, isosulfan blue dye was injected in periareolar orientation at 4 points as a dual agent for SLNB (Fig. 1). All procedures were carried out by the same certified breast surgeons familiar with the ARM and TAD technique with triple mapping via an axillary incision oriented at the inferior border of the hair-bearing area. The fluorescence of ICG was visualized with the SPY Elite® infrared camera system (Stryker Technologies, Inc.) and via a handheld dual opto-nuclear probe (Euoroprobe3) after the ARM procedure was conducted. The camera was focused on the axillary cavity just after incising the clavipectoral fascia but before proceeding further dissection. This step was repeated after TAD. The TAD was carried out via merging the dual agent SLNB with the excising of the clipped node. The clipped node was visualized via intraoperative sonography and excised in each case without failure. Nodes were designated as sentinel nodes if they were radioactive (hot) and/or blue colored. The ex-vivo imaging was performed on the resected nodal specimen to determine the presence of any fluorescence (ICG) or green discoloration. The intensity of the fluorescence was evaluated qualitatively (visual) and quantitatively (SPY camera and dual opto-nuclear probe). Any node with fluorescence was deemed as ICG positive ARM node. The lymphatic drainage after the completion of axillary staging was imaged again via both an infrared camera system and hand-held probe and the axilla was inspected for the presence of ICG positive ARM nodes and lymphatics. Intraoperative frozen section analysis was employed to evaluate SLNs and clipped nodes. The intercostobrachial nerve was routinely spared in all patients during the procedures, as an institutional routine clinical practice. Axillary dissection was performed according to standard guideline criteria. Routine clinical follow-up was pursued for all patients.

### 2.3. Statistical analysis

All outcome summary statistics were analyzed. Descriptive statistics were used as means with standard deviations, and medians with range, whenever appropriate. Categorical variables including clinical characteristics, demographics, and outcome data were summarized via percentages and frequency. Data were analyzed using SPSS Statistics for Windows, version 23.0.

## 3. Results

Fifty-two patients underwent TAD and ARM procedures, 12 of whom had axillary node dissection. The mean age of the was 48.7 years (range; 28–67) and median follow-up was 44 months (IQR 32–56). Baseline characteristics of the patients are demonstrated in Table 1.

**Table 1:** Baseline characteristics.

Characteristics	Frequency (%)
Age, yr	
Mean (range)	48.7 ± 8.3
Tumor size, cm (after NST)	
Mean (range)	1.6 (0–2.8)
Tumor size after NST (%)	
<1 cm	27 (51.9)
1–2 cm	12 (23.0)
2–3 cm	8 (15.4)
>3 cm	5 (9.6)
Bloom-Richardson Grade (%)	
Grade 1	16
Grade 2	28
Grade 3	8
Estrogen positive (%)	25 (48.07)
Progesteron positive (%)	21 (40.38)
HER-2 neu positive (%)	35 (67.3)
Triple negative (%)	9 (17.3)

No adverse reaction was seen due to tracing agents. A median of 4 (IQR 1-9) SLNs was excised per patient.

The overall identification rate of ARM-nodes by means of ICG technique was 86.5% (45 out of 52 patients). Overall crossover of ARM nodes with SLNs (including TAD) was determined in 19 (36.5%) patients (Table 2). The ICG intensity was found to be higher in both hot and blue SLNS (8 out of 18 ICG positive cases, 44.4%). ICG fluorescence were visualized in all 18 ICG positive cases via both SPY Elite® infrared camera system (Stryker Technologies, Inc.) and handheld dual opto-nuclear probe (Euoroprobe3) without any difference regarding the device performance.

**Table 2:** SLN and ARM nodes identification.

SLN identification (TAD) N (%)	52 (100)
Median number of SLNs (range)	4 (1–9)
Number of patients, (%)	
Number of lymph-nodes involved after NST	
0	40 (76.9)
1–3	8 (15.4)
4–9	4 (7.8)
SLN = ARM node (%)	19 (36.5)
TAD node = ARM node (%)	5 (9.6)
Positive node = ARM node	3 (5.8)

ARM = axillary reverse mapping, NST = neoadjuvant systemic therapy, SLN = sentinel lymph node, TAD = targeted axillary dissection.

45 (86.5%) of the 52 patients had at least 1 hot or blue SLN identified intraoperatively. Of these, 32 (61.5%) cases had hot SLNs, 22 (42.3%) had hot and blue, 8 (15.4%) had hot/blue/fluorescent, 4 (7.7%) had blue/fluorescent, 6 (11.5%) had hot/fluorescent and 7 (13.5%) had only clipped nodes (Table 3). In 12 out of 52 patients had metastatic SLNs determined via frozen section or permanent histopathologic analysis and underwent level 1 to 2 ALND. Metastatic nodes were clipped nodes in 5 and ARM nodes in 3 cases. Clipped node was ARM node in 5 (9.6%) patients and positive in all 3 cases. In 3 of 52 patients (5.7%), metastatic SLNs were hot or blue but fluorescent which predicts metastatic involvement of the ARM-nodes (Table 4).

**Table 3:** SLN node characteristics.

SLN	Hot or blue	Hot and blue	Hot/blue/fluorescent	Hot/fluorescent	Blue/fluorescent	Only clip w/o other tracers
Patients (N)	45	22	8	6	4	7
Patients (%)	86.5	42.3	15.38	11.53	7.69	13.46

SLN = sentinel lymph node.

**Table 4:** Metastatic SLN characteristics.

Metastatic nodes	Hot	Blue	Hot and blue	Hot or blue but Fluorescent	Clipped + fluorescent	Clipped
Patients (N)	9	8	5	3	3	2
Patients (%)	17.3	15.4	9.6	5.7	5.7	3.8

SLN = sentinel lymph node.

After TAD completed, imaging of the axilla revealed fluorescent nodes in 12 patients (23.1%) and drainage in 32 patients (61.5%). Whole breast irradiation was administered to all patients with breast-conserving surgery. Irradiation fields were designed individually regarding the tumor size and number of axillary nodes involved/nodal involvement after NST, while level I and II axillary regions were radiated irrelevant to the pathologic response in each case of confirmed axillary metastasis before NST. During follow-up period, systemic metastasis (3 patients), ipsilateral axillary recurrence (1 patient) and ipsilateral breast recurrence (1 patient), were identified as unfavorable outcomes. Three out of 52 patients developed slight lymphedema within 3 years that relieved via physiotherapy, none of whom had undergone ALND or fluorescent SLNs during surgery.

## 4. Discussion

The risk of lymphedema persists after SLNB and aggravates, particularly with the addition of radiotherapy.^[2,18,19]^ Currently, NST is the gold standard method in most of the locally advanced breast cancer patients with axillary metastasis for whom radiotherapy is a must after surgery. ARM has been proposed as a preventive measure to preserve vital anatomic structures during SLNB to avoid lymphedema.^[20–22]^ The value of ARM procedure continues to rise as lymphedema is the worst consequence regarding quality-of-life issues, especially for high-risk patients and 1 of the most dreaded complications of axillary staging for which prevention is absolutely better than cure.^[23,24]^ ICG based fluorescence imaging has been reported to visualize arm draining nodes in breast cancer previously.^[16,25,26]^ Bedrosian et al investigated ARM using isosulfan blue dye in 30 patients with biopsy proven axillary metastatic nodes following NST and detected arm draining nodes in 50% of the patients via this technique, only 2 of whom harvested metastatic disease.^[27]^ In the current study, ICG was injected into the proximal arm and isosulfan blue dye with technetium were injected into the periareolar region circumferentially for breast SLN mapping. Overall, however, a slightly higher number of nodes were hot, blue, and fluorescent than hot or blue and fluorescent, indicating the importance of dual agent mapping. Moreover, the metastasis was found to be higher in nodes positive for triple mapping. The crossover between SLN and ARM nodes was detected in 19 out of 52 cases for whom sentinel nodes were also radioactive and blue in 8 patients. This crossover rate is higher than the literature.^[7,28]^ Foster et al reported a crossover rate of 8.7% with the dual agent ARM procedure, however, a rate of %34.8 was also mentioned when the case of faint overlap with both agents and ICG alone was counted, similar to the rate of 36.5% in the presented study demonstrating the sensitivity of ICG for determining ARM nodes superior to blue dye.^[16]^ Taken together, simultaneous ARM and sentinel node mapping with triple tracers after NST successfully demonstrated the persistence of lymphatic drainage from the breast, which made SLNB possible and extensive anatomic connections between the lymphatic pathways of the arm and the breast which could be determined via ICG mapping. The continuity of the axillary drainage from the arm was confirmed in 44 out of 52 patients in the current study and none developed clinically significant lymphedema at the time of more than 3 years follow-up.

One of the most important issues to be emphasized about the presented study is the rate of metastasis in ARM node after NST which is 5.7% and all cases were luminal-like type tumors. Contrary to the data of Bedrosian et al, reporting this rate as 18.8%, the lower rate of nodal involvement after systemic therapy merits consideration, particularly in the era of precision medicine, leading higher than 80% of pathologic complete response rates after NST for selected patients with triple negative or HER-2 positive breast cancer under dual agent blockage.^[27]^ In the presented series, 67.3% of the cases were HER-2 positive and had dual agent blockage in the neoadjuvant setting. Accordingly, it is rational to consider preserving ARM nodes, at least in this subpopulation without impairing oncologic safety.

Regarding the technical details, on inspection of the axilla following SLNB procedure, the integrity of the lymphatic drainage could only be confirmed via visualization of green and fluorescent nodes and lymphatics through the axilla. To have the ability to assess this integrity, all lymphatics should be clipped or sutured but not cauterized during SLNB. Otherwise, ICG leakage would cause catastrophic consequences in terms of diagnostic accuracy. The complete visualization of the axillary operative field via infrared camera system is usually difficult and even not possible due to the limited exposure through a small incision and the anatomic concavity. In the current study, a dual opto-nuclear probe was used for ICG detection in conjunction with a infrared camera, enabling to visualize the lymphatic drainage from the injection site in the upper arm up to the posterior medial part of the pectoralis minor muscle confirming the lymph flow beyond the boundaries of SLNB. The higher crossover rate in the presented study might be attributed to this technical detail which detects even the fainted fluorescence in the nodes evaluated, as well. Therefore, it is rational to presume that lymphedema is not only a function of the number of lymph nodes removed or whether ALND is performed or not, but the absence of lymphatic flow from the arm through the axilla after axillary staging intervention is completed. Moreover, the results of the presented study demonstrate that when level I-II ALND dissection is done meticulously to spare the lymphatics, radiotherapy might account for as the primary etiologic factor for lymphedema. Therefore, determining the patient subgroup who can be spared from radiotherapy for clinically and radiologically negative patients is of paramount importance that would provide a more sensitive and individualized approach to axillary lymph nodes which are only indicators in the name of staging, but do not dominate or govern distant metastases in the prognosis of the disease.

There are several limitations of the presented study including single center experience, limited sample size, and relatively short follow-up period for lymphedema. However, the higher crossover rate and lower rate of metastasis in ARM nodes after NST merit consideration.

In summary, our study demonstrated that dual agent mapping for SLNB and ICG for ARM is a valuable technique after NST to determine the required number of SLNs for oncologic safety, to preserve axillary drainage and to prevent lymphedema. The concerns regarding the accuracy of the SLNB technique together with anxiety of the risk of lymphedema could be reduced via triple mapping.

## 5. Conclusion

The crossover rate in the current study is more than previously reported after NST. The employment of a dual opto-nuclear probe with ICG infrared camera is superior in the identification of arm lymphatics and nodes. The higher observed rate of overlap might partially explain why more patients develop clinically significant lymphedema after NST even after SLNB alone. The triple mapping provides valuable data regarding the competency of lymphatic drainage and would have the potential to serve selecting patients for lymphovenous by-pass procedures at the index procedure. NST reduces the metastatic involvement of the ARM nodes. However, conservative axillary staging with sparing ARM nodes after NST necessitates further studies with larger sample size and longer follow-up.

## Author contributions

**Conceptualization:** Ilhan Tasdoven, Rabiye Uslu Erdemir, Burak Bahadir, Cakmak Guldeniz Karadeniz.

**Data curation:** Rabiye Uslu Erdemir.

**Formal analysis:** Rabiye Uslu Erdemir.

**Methodology:** Burak Bahadir.

**Resources:** Cakmak Guldeniz Karadeniz.

**Writing – original draft:** Ilhan Tasdoven, Hakan Balbaloglu, Cakmak Guldeniz Karadeniz.

**Writing – review & editing:** Ilhan Tasdoven, Hakan Balbaloglu, Rabiye Uslu Erdemir, Cakmak Guldeniz Karadeniz.
